# Network-based integration of cross-dataset proteomic profiles using fold-change directionality

**DOI:** 10.1093/bioadv/vbag321

**Published:** 2026-10-23

**Authors:** Manaka Nishizaki, Norie Araki, Shin Kawano

**Affiliations:** Graduate School of Frontier Engineering, Kitasato University, Sagamihara, Kanagawa, 252-0373, Japan; Graduate School of Medical Sciences, Faculty of Life Sciences, Kumamoto University, Kumamoto, Kumamoto, 860-8556, Japan; Graduate School of Frontier Engineering, Kitasato University, Sagamihara, Kanagawa, 252-0373, Japan; Database Division for Life Science, BioData Science Initiative, National Institute of Genetics, Research Organization of Information and Systems, Kashiwa, Chiba 277-0871, Japan

## Abstract

**Motivation:**

The rapid expansion of proteomic data has created new opportunities for large-scale integrative analyses. However, substantial variability across platforms, experimental designs, and processing pipelines limits direct quantitative comparisons among studies. Differential proteomic changes between conditions are often considered to be more reproducible than absolute abundances and may therefore provide a robust basis for cross-dataset integration. However, the systematic ability of differential change-based approaches to capture biologically meaningful relationships across heterogeneous datasets remains unclear.

**Results:**

We developed a differential-change framework and applied it to public proteomic datasets. Pairwise contrasts were defined as differential proteomic profiles, and the concordance of up- and down-regulated proteins was quantified using odds ratios. Significant profile pairs were visualized as an integrated network. The treatment of anti-cancer drug doxorubicin vs control (MCF-7) comparison emerged as a central hub, with breast cancer proteome profiles clustering around it and associating with tumor stage (*P *= 0.0386). Enrichment analysis revealed overrepresentation of lipid- and cholesterol-related pathways.

**Availability and implementation:**

The source code for proteome network integration is available at https://github.com/manakanishizaki/proteome-network-integration.git

## 1 Introduction

Proteomics aims to comprehensively characterize proteins in biological systems, including their abundances, modifications, and interaction networks, and plays a key role in elucidating cellular mechanisms, disease processes, and new biomarkers (Aebersold and Mann 2016). Advances in mass spectrometry (MS) have enabled simultaneous quantification of thousands of proteins with high sensitivity and accuracy (Birhanu 2023). Concurrently, large volumes of proteomic data generated across laboratories and analytical platforms have been deposited in public repositories. Major resources include PRIDE (Perez-Riverol *et al.* 2025), MassIVE (Choi *et al.* 2020), and jPOST (Okuda *et al.* 2025), members of the ProteomeXchange Consortium (Deutsch *et al.* 2026), as well as the Clinical Proteomic Tumor Analysis Consortium (CPTAC) (Edwards *et al.* 2015), which provides extensive cancer proteomics datasets. Additionally, initiatives such as quantMS (Dai *et al.* 2024) provide standardized workflows for reanalyzing public datasets and disseminating results. The increasing availability of these resources creates new opportunities to reuse proteomic data across studies and to identify biological relationships that may not be apparent from individual datasets. However, effective cross-study integration remains challenging because these datasets are generated using diverse experimental designs, analytical platforms, and data-processing workflows.

Two principal quantification strategies are commonly employed in MS-based proteomics: label-free quantification (LFQ) (Bantscheff *et al.* 2012) and labeling-based approaches (Wang *et al.* 2024). LFQ estimates relative protein abundance from peptide intensities or spectral counts (Bantscheff *et al.* 2012) without using chemical tagging, facilitating analysis of large cohorts. Labeling-based methods, such as tandem mass tag (TMT) (Thompson *et al.* 2003) and isobaric tag for relative and absolute quantitation (iTRAQ) (Chong *et al.* 2006, Rauniyar and Yates 2014) combine multiple samples within a single MS run, reducing run-to-run variability and enabling precise relative quantification. Consequently, quantitative proteomic datasets available for reuse are generated using diverse experimental and quantification strategies, and their numerical abundance measurements cannot necessarily be treated as directly comparable across studies.

Beyond differences in quantification strategy, technical variability remains a major barrier to cross-study comparison. Differences in instrumentation, sample preparation, labeling strategies, and computational pipelines restrict direct comparability of proteomic measurements (Bantscheff *et al.* 2012). Protein abundance measurements can also be affected by study-specific technical and batch effects, complicating data reuse and cross-platform integration. Such variability impedes large-scale meta-analyses and limits the full utilization of rapidly expanding public proteomic resources.

Ratio-based quantitative strategies have also been shown to improve cross-platform reproducibility in multi-omics studies (Zheng *et al.* 2024). Accordingly, differential proteomic changes between experimental conditions may be less sensitive to study-specific quantitative scales and systematic effects than direct comparisons of protein abundance values. In metabolomics, iDMET introduced a data-driven, network-based strategy for integrating heterogeneous studies by comparing differential metabolomic profiles pairwise rather than directly merging absolute metabolite levels (Matsuta *et al.* 2022). This approach enabled exploration of relationships among independently generated datasets even when the underlying quantitative measurements were not directly comparable. Together, these observations suggest that differential profiles may provide an alternative basis for integrating heterogeneous omics datasets. However, whether this strategy can be extended to heterogeneous proteomic datasets, which likewise differ in experimental design, analytical platform, processing workflow, and protein coverage, remains unclear.

In the present study, we extended the concept of differential-profile integration, previously applied in metabolomics, to heterogeneous proteomic datasets. We hypothesized that differential protein-expression directionality could provide a practical basis for exploratory cross-study integration and that biologically related experimental conditions would exhibit concordant patterns of up- and down-regulated proteins across independently generated datasets. We applied this directionality-based framework to publicly available proteomic datasets from quantMS and CPTAC and constructed a condition-level network based on pairwise concordance of differential proteomic profiles. We then evaluated whether the resulting cross-study relationships were robust to analytical choices and biologically interpretable by examining protein-level fold-change concordance, clustering of related experimental conditions, associations with clinical variables, and functional enrichment. Thus, rather than developing a predictive or classification model, our aim was to evaluate the applicability and limitations of differential protein-expression directionality as a data-driven strategy for exploratory integration of heterogeneous public proteomic datasets.

## 2 Methods

### 2.1 Data sources

We used publicly available, label-based quantitative proteomic datasets to systematically evaluate concordance in the direction of protein expression changes across conditions and to analyze the resulting network structure and its biological and clinical relevance.

We used two large-scale proteomics repositories: quantMS (Dai *et al.* 2024) and CPTAC (Edwards *et al.* 2015), both providing labeled quantitative MS datasets. From quantMS, we retrieved labeled-quantification projects, totaling 36 projects and 2371 experimental conditions. From CPTAC, we obtained 49 TMT-based projects comprising 5260 conditions.

Here, an “experimental condition” refers to a distinct condition or comparison label associated with a protein-level quantitative profile (e.g. tumor versus control or drug treatment versus control), rather than an individual biological sample or replicate. Thus, the reported numbers represent the number of distinct quantitative condition profiles available in each resource and not the total number of individual samples.

A complete list of all project identifiers is provided in Table S1.

### 2.2 Preprocessing and quality control

Prior to analysis, protein identifiers from quantMS and CPTAC were standardized (Fig. 1A) and subjected to quality control. In quantMS, entries containing multiple protein identifiers separated by semicolons were expanded into separate rows. During this expansion, all quantitative values associated with the original entry were retained for each resulting protein identifier; only the protein identifier field was changed. No additional aggregation was performed during this expansion step. Entries labeled DECOY or CONTAMINANT were removed. For UniProt-formatted identifiers (sp|Accession|ProteinName), the final ProteinName component following the last vertical bar was retained, and species suffixes (e.g. _HUMAN) were removed to standardize notation.

**Figure 1:**
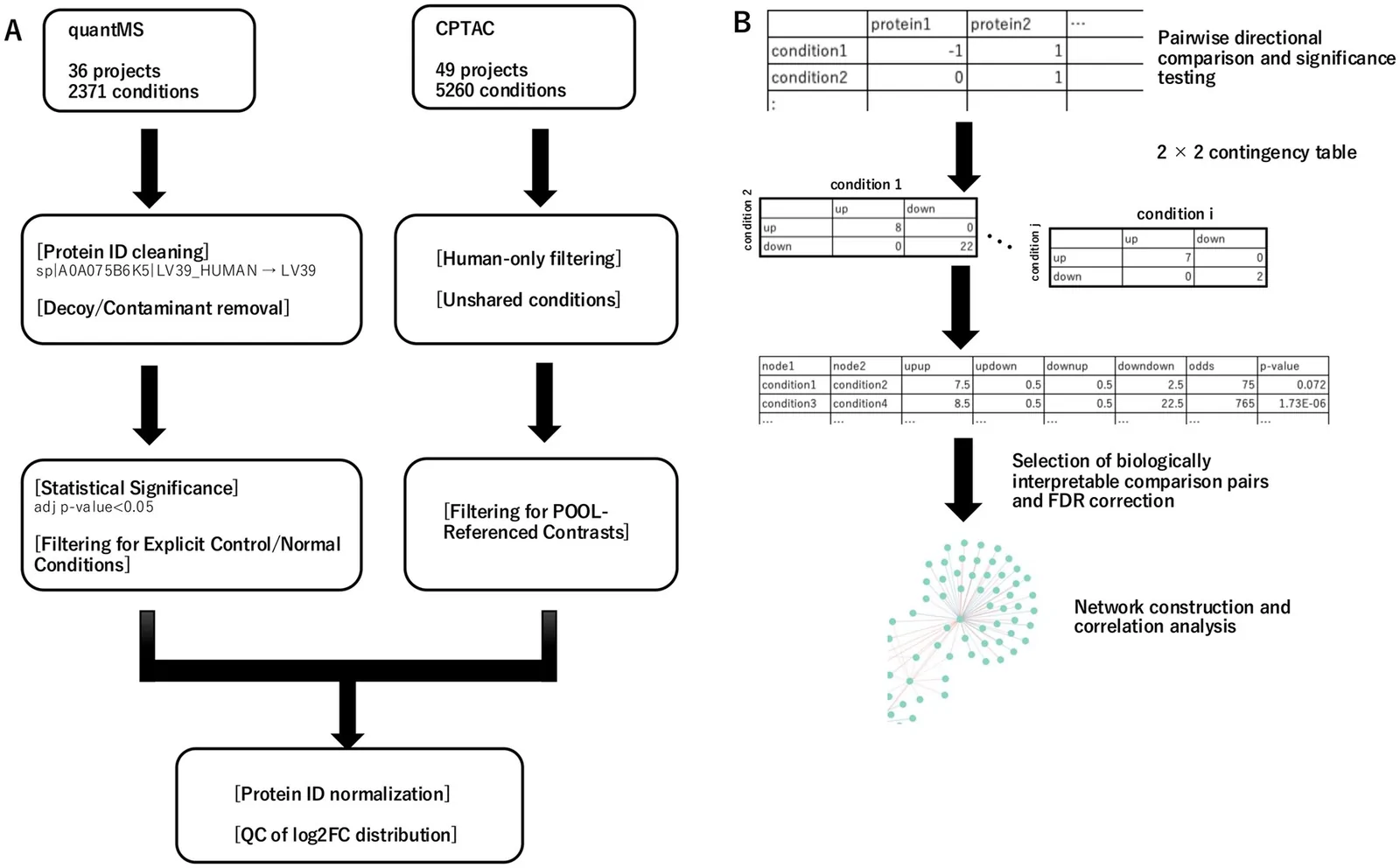
Workflow for identifier normalization and network construction. (A) Overview of proteomic data collection and preprocessing. Proteomic datasets were obtained from quantMS (36 projects and 2371 experimental conditions) and CPTAC (49 projects and 5260 experimental conditions). In quantMS, protein identifiers were cleaned and standardized, including removal of decoy and contaminant entries. In CPTAC, analyses were limited to *Homo sapiens* proteins and conditions labeled “unshared.” After dataset-specific filtering, identifiers were normalized across resources, and the log2 fold-change (log2FC) distributions were evaluated prior to downstream analysis. (B) Differential-change-based integration and network construction. For each condition, differential protein changes were encoded as directional signatures. Pairwise comparisons were performed to assess concordant and discordant directional changes, followed by statistical testing. Biologically interpretable pairs were selected using a false discovery rate (FDR) threshold and used to construct a network, enabling correlation analysis and identification of relationships across studies.

For CPTAC, only proteins annotated as *Homo sapiens* in the Organism field were included, and analyses were restricted to conditions labeled “unshared” to avoid interference from shared peptides and ensure more reliable quantification. To define significance thresholds, log_2_ fold-change (log2FC) distributions were examined across all conditions. The 5th–95th percentile range of the overall log2FC distribution was −1.03 to 0.92, indicating that most observed values were within approximately ±1. Based on this distribution, |log2FC| ≥ 1, corresponding to at least a twofold change, was used as the threshold for defining directional protein changes. In quantMS, log2FC and multiple testing-adjusted *P-*values were available and used for filtering (|log2FC| ≥ 1 and adjusted *P* < 0.01). Conversely, because CPTAC data lacked *P*-values, only log2FC thresholds were applied. To assess whether this asymmetric filtering strategy influenced the resulting network topology, we additionally performed a sensitivity analysis in which the adjusted *P*-value criterion was omitted from quantMS and both quantMS and CPTAC were filtered using the same fold-change criterion (|log2FC| ≥ 1). All subsequent directional comparison, significance testing, contrast selection, and network construction procedures were performed using the same criteria as in the primary analysis.

### 2.3 Pairwise directional comparison and significance testing

Directional concordance of protein expression changes was evaluated for all condition pairs using directionality tables derived from quantMS and CPTAC. Proteins were classified as upregulated (log2FC ≥ 1) or downregulated (log2FC ≤ −1), and 2 × 2 contingency tables were constructed for each pair (Fig. 1B). Odds ratios and statistical significance were computed from upregulation and downregulation patterns. When one or more cells of a 2 × 2 contingency table contained zero, a Haldane–Anscombe continuity correction was applied by adding 0.5 to all four cells before calculating the odds ratio. Chi-square tests were used to assess independence of expression direction. Analyses were conducted for all condition pairs within each dataset, followed by extraction of biologically interpretable contrasts and application of a false discovery rate (FDR) correction.

### 2.4 Selection of biologically interpretable comparison pairs and FDR correction

Among all tested pairs, only biologically interpretable contrasts with consistent reference conditions were retained. In CPTAC, contrasts representing changes relative to pooled reference samples were selected, whereas in quantMS, contrasts with explicit references (e.g. control or normal) were included. Analyses were restricted to pairs from different projects; CPTAC projects reanalyzed in quantMS were excluded to preserve independence.

Because the biological reference conditions differed between quantMS and CPTAC, cross-resource comparisons were interpreted as comparisons of relative differential patterns rather than as direct comparisons of equivalent biological baselines.

Specifically, the analysis evaluated concordance in the direction of protein changes (up- or downregulation) relative to the reference defined within each contrast; it did not assume equivalence of the underlying reference states or directly compare fold-change magnitudes between resources.

*P*-values obtained from directional concordance testing were adjusted using the Benjamini–Hochberg FDR procedure, and pairs with FDR < 0.01 were deemed significant.

### 2.5 Network construction and correlation analysis

Networks were generated from filtered contrasts in quantMS and CPTAC. Nodes represent comparison conditions, and edges connect pairs showing significant directional concordance in protein expression (FDR < 0.01). Network construction and visualization were performed using Cytoscape (Shannon *et al.* 2003). To examine whether strong directional concordance identified by the network also corresponded to agreement in the underlying continuous fold-change values, the condition pair with the highest odds ratio was selected for correlation analysis. Log2FC values for proteins quantified in both conditions were extracted, Pearson correlation coefficients were calculated, and scatter plots were generated for all commonly quantified proteins and for proteins meeting the predefined differential-change threshold, to evaluate agreement between conditions and consistency with network-derived association strength.

### 2.6 Hierarchical clustering of expression profiles across conditions

To assess condition similarity, log2FC values for proteins in the experimental conditions connected to the quantMS-derived condition of interest were extracted from the corresponding project-level datasets and integrated into a protein-by-condition matrix. The extracted datasets were merged by protein identifier using an inner join, such that only protein identifiers represented across all included project-level datasets were retained. Infinite values were treated as missing. Because the integrated matrix consisted of log2FC values rather than protein abundance or intensity measurements, missing values could not be directly interpreted as low-abundance measurements. In the primary analysis, missing log2FC values were replaced with zero, which represents no differential change and does not assign either an increase or decrease to the missing entry. Similarity among conditions and proteins was evaluated using hierarchical clustering with Euclidean distance and Ward’s linkage, applied to rows (proteins) and columns (contrasts) to visualize expression patterns.

To assess whether zero replacement influenced the resulting clustering structure, two sensitivity analyses were additionally performed. First, missing values were replaced with the median log2FC calculated separately for each experimental condition (column). Second, the clustering analysis was repeated after excluding proteins containing one or more missing values across the included conditions, thereby eliminating the need for missing-value replacement. All three analyses used the same Euclidean distance, Ward’s linkage, and cluster-number settings.

Cluster assignments obtained using zero replacement, median imputation, and complete-case analysis were compared using the adjusted Rand index (ARI), separately for protein (row) and condition (column) clustering.

### 2.7 Association with clinical variables and functional enrichment analysis

Associations between contrast clusters and clinical categorical variables were evaluated using chi-square tests. Analyses were conducted using CSV files containing patient clinical annotations, with the following variables considered: ethnicity, race, primary diagnosis, tissue or organ of origin, tumor grade, tumor stage, disease type, and primary site. Samples labeled “Not Reported” or “Unknown” were excluded.

To support biological interpretation of clustered proteins, functional enrichment analysis was performed via Metascape (Zhou *et al.* 2019) based on Gene Ontology (GO) Biological Process terms (Ashburner *et al.* 2000) and Kyoto Encyclopedia of Genes and Genomes (KEGG) pathway (Kanehisa and Goto 2000) annotations. Subtype-specific analyses were not performed because certain cancer subtype annotations reported in the original CPTAC publications were unavailable in the clinical datasets analyzed.

## 3 Results

### 3.1 Establishment of the study workflow

The overall workflow for cross-dataset proteomic integration is summarized in Fig. 1. Proteomic data from quantMS and CPTAC were subjected to dataset-specific preprocessing and protein-identifier harmonization, followed by pairwise comparisons of differential protein-expression directionality. Significant directional relationships between experimental conditions were subsequently represented as a condition-level network for downstream evaluation of cross-study relationships.

### 3.2 Quality control and significant proteins

The analyzed projects encompassed diverse contrasts, including drug-treated versus control samples and tumor versus normal tissues. The quantMS and CPTAC datasets identified 14 508 and 15 328 proteins, respectively, with 6303 proteins detected in both resources (Fig. 2A).

**Figure 2:**
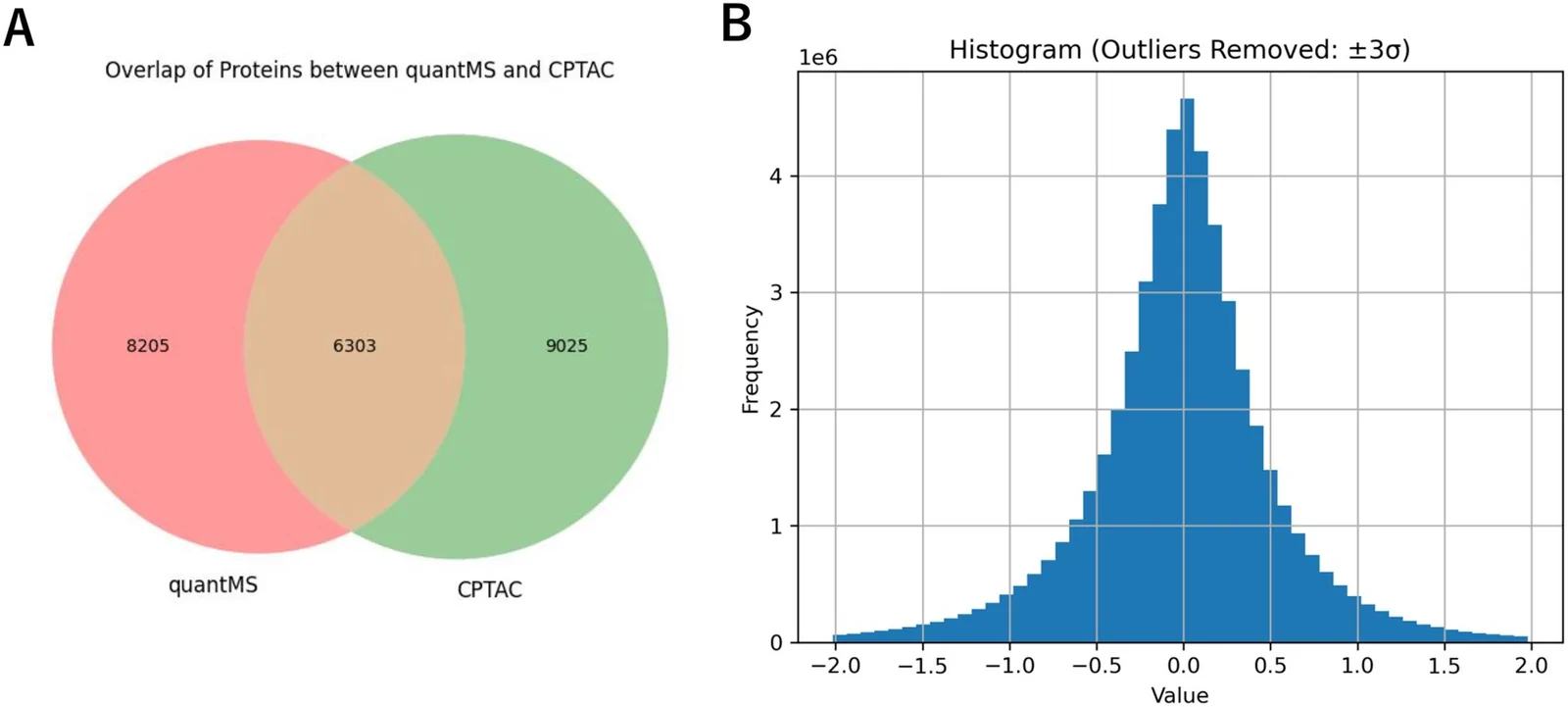
Dataset statistics. (A) Overlap of proteins identified in quantMS and CPTAC. Venn diagram shows overlap between the 14 508 and 15 328 proteins detected in quantMS and CPTAC, respectively, with 6303 shared proteins identified. (B) Distribution of log2FC values in quantMS and CPTAC. Histograms show log2FC distributions across all datasets. Values exceeding ±3 standard deviations from the mean were excluded from visualization to improve clarity but retained in downstream analyses and quantitative datasets.

To characterize differential expression, percentile-based metrics were used to summarize global fold-change distributions. Across projects, most log2FC values fell within ±1 (5th–95th percentile: −1.03 to 0.92; Fig. 2B and Fig. S1). Thus, although the two resources differed substantially in overall protein coverage, their global log2FC distributions were similarly centered around relatively small differential changes, with larger changes occurring in a smaller subset of proteins.

### 3.3 Initial network construction and observations

To compare fold-change directionality across quantMS and CPTAC, only contrasts with consistent, interpretable reference conditions were retained. In CPTAC, contrast annotations were heterogeneous: many contrasts not explicitly formatted as “A/B” were inferred to represent changes relative to pooled references (POOL), whereas explicitly annotated “A/B” comparisons often involved non-POOL references, reference–reference comparisons (e.g. QC–QC, bridge–bridge, or CR–CR), or sample–sample contrasts lacking a shared reference. Because these formats were unsuitable for directional analysis, they were excluded using predefined criteria.

After filtering, 463 CPTAC contrasts were removed, leaving 4797 POOL-referenced contrasts. In quantMS, contrasts lacking explicit reference conditions were excluded, yielding 28 contrasts for downstream analysis. The resulting initial condition-level network comprised 639 nodes and 1040 edges (Fig. S2).

The highest odds ratio was observed between “PDC000126_normal–ovarian serous carcinoma” (quantMS-derived) and “PDC000125_CPT0026540001 Unshared Log Ratio” (CPTAC-derived). PDC000126 represents a reanalysis of the CPTAC UCEC Discovery Study phosphoproteome, whereas PDC000125 corresponds to a proteome dataset from the same CPTAC study (Dou *et al.* 2023). Thus, the strongest directional concordance in the initial network was observed between datasets originating from the same biological study but processed through different analytical workflows and representing different molecular layers. Because this association did not represent an independent cross-study relationship, CPTAC projects reanalyzed in quantMS were excluded from subsequent analyses.

### 3.4 Network established after removal of reanalyzed CPTAC projects

After excluding CPTAC projects that had been reanalyzed on quantMS, the refined network contained 287 nodes and 357 edges (Fig. 3). Each edge represented a condition pair with significant directional concordance based on the chi-square test. The network topology revealed a subset of highly connected nodes. These hub nodes corresponded to experimental conditions exhibiting directional concordance with multiple other conditions and were frequently associated with relatively large differential changes. Major hub nodes and their degrees are summarized in Table 1.

**Figure 3:**
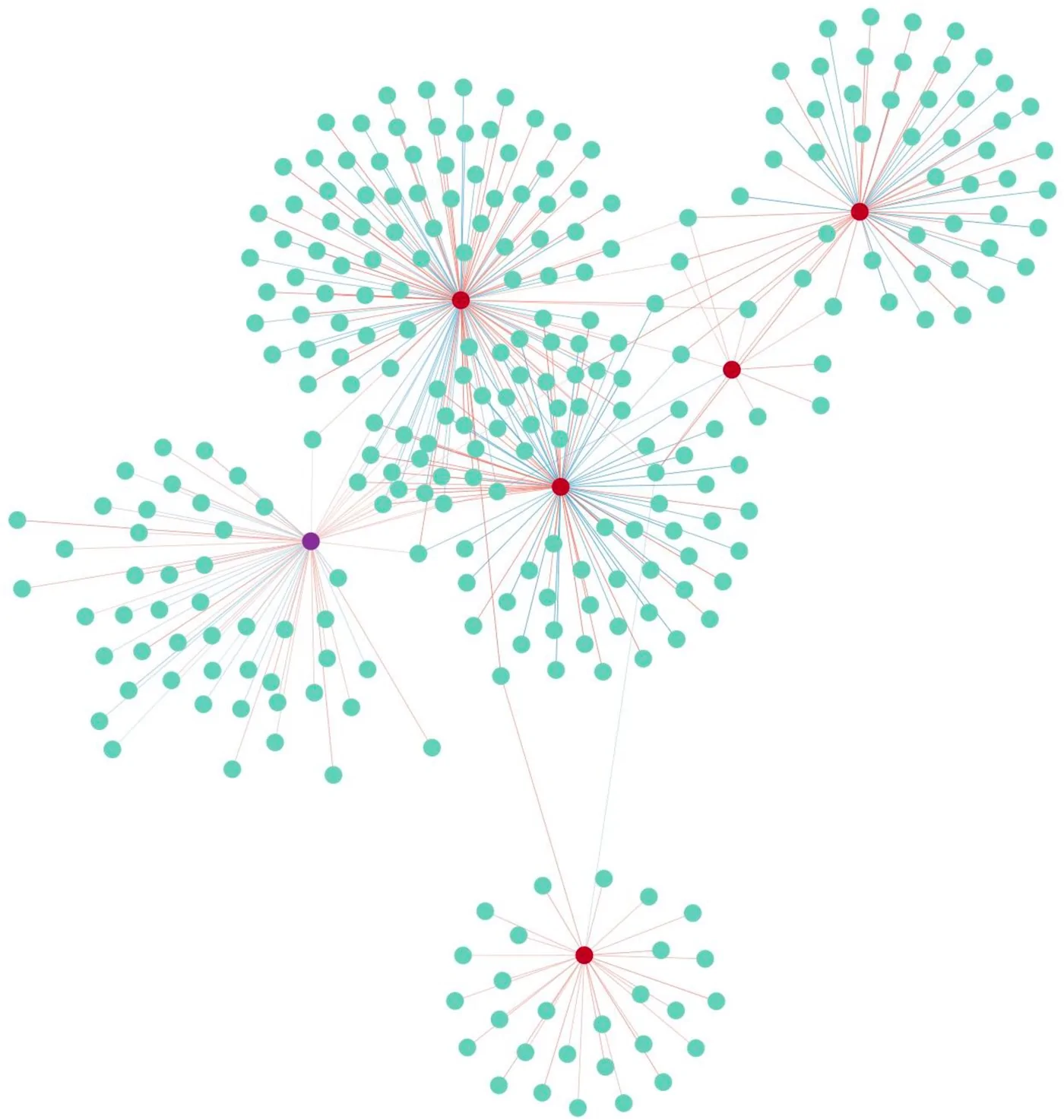
Integrated network constructed after excluding CPTAC projects reanalyzed by quantMS. The network was generated after removing CPTAC-derived projects reanalyzed by quantMS and consists of 287 nodes and 357 edges. Edges represent condition pairs with statistically significant associations based on chi-square tests with FDR correction (*q* < 0.01). Edge colors indicate the direction of association (red for positive and blue for negative). Hub nodes are highlighted in red, and the node of particular interest (PXD030881_anticancer doxorubicin–control) is highlighted in blue.

**Table 1:** Hub nodes identified in the integrated proteomic network derived from quantMS and CPTAC data.

Project ID	Node	Comparison type	Cancer type	Edges	Reference
**PXD002137**	Adenoma-normal	Tumor vs normal	Adenoma	11	Wiśniewski *et al.* 2015
**PXD004683**	Normal-squamous cell carcinoma	Tumor vs normal	Squamous cell carcinoma	80	Stewart *et al.* 2017
**PXD004873**	Hepatocellular carcinoma-normal	Tumor vs normal	Hepatocellular carcinoma	54	Zhu *et al.* 2019
**PXD012574**	High grade ovarian serous adenocarcinoma-normal	Tumor vs normal	Ovarian serous carcinoma	119	Persi *et al.* 2019
**PXD030881**	Anticancer drugs doxorubicin-control	Drug treatment vs control	Breast cancer (MCF-7)	63	Cha *et al.* 2022
**PXD030881**	Anticancer drugs paclitaxel-control	Drug treatment vs control	Breast cancer (MCF-7)	30	Cha *et al.* 2022

Each node represents a comparison between control and treated conditions within the same biological context, e.g. tumor vs. normal tissue or drug treatment vs. control. The number of edges indicates the node degree, reflecting the number of significant correlations with other nodes in the network. Associated cancer types and project identifiers are provided.

To evaluate the influence of the asymmetric filtering strategy between quantMS and CPTAC, we reconstructed the network after omitting the adjusted *P*-value criterion from quantMS and applying the fold-change threshold alone to both resources. The resulting network expanded to 1179 nodes and 2275 edges. Despite this increase, 278 of the 287 nodes (96.9%) and 307 of the 357 edges (86.0%) in the primary network were retained. Major hub conditions identified in the primary analysis, including the doxorubicin–control condition, also remained highly connected. Thus, removing the adjusted *P*-value criterion substantially increased network size while largely preserving the principal topology of the original network.

### 3.5 Correlation analysis of the highest odds-ratio pair

For the pair with the highest odds ratio, correlation analysis was performed between ovarian cancer (PXD012574_high grade ovarian serous adenocarcinoma–normal) and uterine cancer (PDC000439_CPT0127980003 Unshared Log Ratio) using the log2FC values of commonly quantified proteins (Fig. 4).

**Figure 4:**
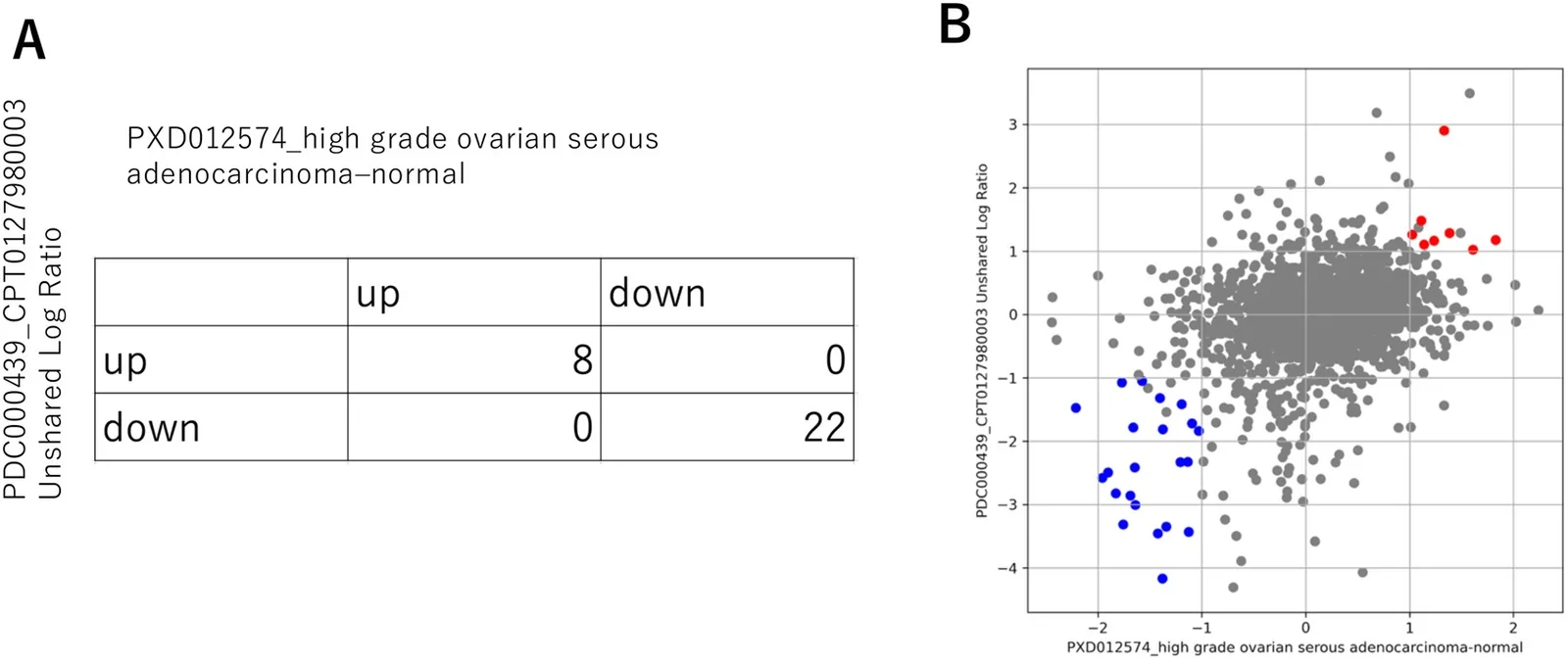
Comparison of log2FC values for protein expression between ovarian and uterine cancers. (A) Contingency table (2 × 2) summarizing proteins with concordant increases or decreases across conditions. Association was assessed using the chi-square test, yielding a log_2_ odds ratio of 7.65 and a *P-*value of 1.7 × 10^−6^. (B) Protein log2FC compared between ovarian cancer (PXD012574_high grade ovarian serous adenocarcinoma–normal) and uterine cancer (PDC000439_CPT0127980003 Unshared Log Ratio). Proteins exceeding the predefined threshold (|log2FC| ≥ 1) are highlighted: red, upregulated; blue, downregulated.

Cross-tabulation revealed that 8 and 22 proteins were significantly upregulated and downregulated, respectively, under both conditions, whereas no proteins showed significant changes in opposite directions. Across all quantified proteins, the Pearson correlation coefficient was 0.318, indicating moderate overall correlation. In contrast, restricting analysis to significantly altered proteins increased the coefficient to 0.801, demonstrating strong concordance in expression direction.

Although representing distinct tumor types, ovarian and uterine cancers exhibited similar proteomic alteration patterns. This similarity is biologically plausible because both tumors are epithelial in origin and share oncogenic processes, including PI3K/AKT and MAPK signaling, hormone responsiveness, metabolic reprogramming, and cytoskeletal remodeling (Cheaib *et al.* 2015, Aseervatham 2020). These shared molecular features likely underlie the strong directional concordance observed among significantly altered proteins.

### 3.6 Biological interpretations

We systematically evaluated relationships among experimental conditions from multiple proteomics databases using network analysis and hierarchical clustering of log2FC profiles. As a representative case, we examined a hub node corresponding to the breast cancer cell line MCF-7 treated with the anticancer drug doxorubicin (PXD030881_anticancer doxorubicin–control). This condition was selected because it exhibited numerous significant similarities to other conditions and represented a well-defined drug-versus-control contrast.

Hierarchical clustering centered on this doxorubicin–control condition identified one clearly defined major cluster of breast cancer–related experimental conditions, whereas other breast cancer contrasts were more dispersed (Fig. 5). Proteins within the major cluster showed coherent expression patterns, indicating shared molecular features across conditions.

**Figure 5:**
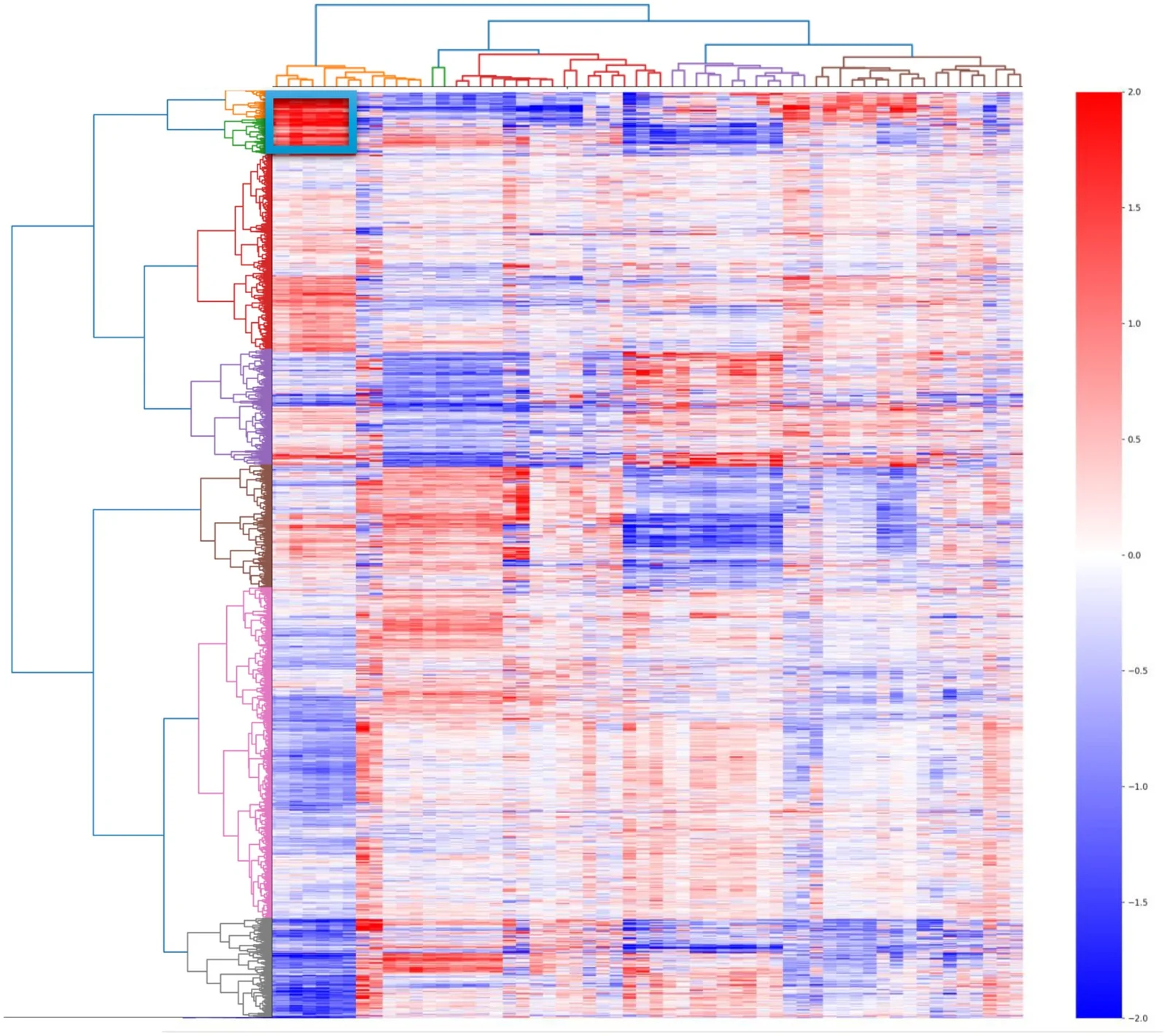
Heatmap of CPTAC-derived experiments associated with the quantMS-derived doxorubicin–control hub. Heatmap summarizes the protein expression patterns of CPTAC-derived experimental conditions linked to the quantMS-derived doxorubicin–control comparison. Proteins (rows) and conditions (columns) were hierarchically clustered, with dendrograms shown on the left and top, respectively. Leftmost condition cluster corresponds to the subset of breast cancer samples that was analyzed further.

To evaluate whether the treatment of missing log2FC values influenced the clustering pattern, we compared the original zero-replacement analysis with column-wise median imputation and complete-case analysis. Protein-level cluster assignments differed among the three approaches (ARI = 0.377 for zero replacement versus median imputation, 0.340 for zero replacement versus complete-case analysis, and 0.513 for median imputation versus complete-case analysis). In contrast, condition-level cluster assignments were identical across all three approaches (ARI = 1.000 for all pairwise comparisons; n = 63 conditions). The complete-case analysis retained 3452 of the 3782 proteins included in the imputed analyses. Thus, although detailed protein-level clustering was sensitive to missing-value treatment, the condition-level cluster structure used for subsequent biological interpretation was preserved across all three approaches.

Integration with clinical metadata indicated that samples in the major cluster tended to have more advanced tumor stages (chi-square test, *P *= 0.0386). Although the effect size was modest, this finding suggests a potential link between proteomic patterns and tumor progression.

Functional enrichment analysis of proteins upregulated in the major cluster revealed significant enrichment of pathways related to lipid metabolism, extracellular matrix (ECM) remodeling, and tissue repair (Fig. S3). These processes have previously been implicated in breast cancer progression and tumor microenvironment remodeling (Zhao *et al.* 2017, Krug *et al.* 2020, Tang *et al.* 2022), supporting the biological relevance of the observed cluster structure.

Collectively, these findings demonstrate that the directional consistency–based network and clustering framework employed in this study can extract biologically and clinically interpretable patterns across heterogeneous proteomic datasets, while the sensitivity analysis further showed that the condition-level clustering underlying these interpretations was robust to the treatment of missing values.

## 4 Discussion

In this study, we extended a directionality-based differential-profile integration concept previously applied to metabolomics to heterogeneous proteomic datasets and evaluated its utility for exploratory cross-study integration. By representing experimental conditions according to the direction of differential protein changes, we constructed a condition-level network across independently generated datasets from quantMS and CPTAC. Hierarchical clustering centered on the doxorubicin–control profile revealed one clearly defined major cluster and several heterogeneous groups, indicating that differential protein-expression directionality can capture coherent condition-level relationships across independent studies despite differences in analytical platforms and data-processing workflows.

Conditions within the major cluster were relatively enriched for advanced tumor stages, with a modest but statistically significant association. Upregulated proteins were enriched for pathways related to lipid metabolism and ECM remodeling, consistent with prior reports on breast cancer biology (Zhao *et al.* 2017, Tang *et al.* 2022), supporting the biological interpretability of the condition-level relationships identified by our analysis. In contrast, downregulated proteins showed modest overrepresentation of core cellular processes, including translation, endoplasmic reticulum protein processing, and DNA replication. This contrast may reflect a greater contribution of condition-associated biological processes among the upregulated proteins, whereas the downregulated proteins were more broadly associated with fundamental cellular functions.

For cross-dataset integration, protein-level changes were defined based on fold-change directionality using a |log2FC| ≥ 1 threshold. This strategy avoids direct comparison of quantitative scales across datasets and may therefore reduce sensitivity to platform-dependent differences in quantitative scale and normalization. Consequently, fold-change magnitude was not explicitly incorporated into network construction or similarity assessment. While this approach may reduce the influence of variability arising from differences in experimental platforms and data-processing pipelines, it may also reduce sensitivity to subtle but consistent changes or biological effects primarily reflected in fold-change magnitude. Thus, the directionality-based representation involves a trade-off between cross-study comparability and retention of quantitative information.

Because the primary analysis applied an adjusted *P*-value criterion to quantMS but not to CPTAC, we additionally evaluated whether this asymmetric filtering strategy affected the network topology. Removing the adjusted *P*-value criterion substantially increased the number of nodes and edges, as expected under the less stringent filtering, while 96.9% of the original nodes and 86.0% of the original edges in the refined network were retained. Major hub conditions, including the doxorubicin–control condition, also remained highly connected. These findings suggest that the adjusted *P*-value criterion primarily constrained network size, whereas the principal network topology and major hubs were largely preserved, supporting the robustness of the main network structure to this analytical choice.

Additional sensitivity analyses further clarified both the robustness and the limitations of the downstream clustering analysis. Because the clustering matrix consisted of derived log2FC values rather than protein abundance or intensity measurements, conventional missing-not-at-random assumptions based on low protein abundance could not be directly applied. Zero was therefore used in the primary analysis to represent no directional change without assigning either an increase or decrease to missing log2FC entries. When the analysis was repeated using column-wise median imputation or complete-case analysis, protein-level cluster assignments showed moderate variation, whereas condition-level cluster assignments were identical across all three approaches (ARI = 1.0 for all comparisons). Thus, although the detailed grouping of individual proteins should be interpreted with caution, the condition-level cluster structure underlying the subsequent biological and clinical interpretations was not dependent on zero replacement. This stability supports the robustness of the principal condition-level relationships, while highlighting greater uncertainty in protein-level clustering.

A further consideration is that the biological reference conditions differed between the two resources. QuantMS contrasts were defined relative to explicit biological controls, whereas the CPTAC contrasts included in this analysis were defined relative to pooled references. Accordingly, a cross-resource network edge should not be interpreted as indicating equivalent biological baselines or directly comparable effect sizes. Rather, it represents concordance in the relative direction of protein changes within each independently defined contrast. This distinction limits the biological interpretation of individual cross-resource edges but is consistent with the exploratory objective of identifying shared differential patterns across heterogeneous datasets.

Several limitations of the present study should be noted. First, integration across proteomic datasets relied on protein identifiers as reported in the original studies. Although efforts were made to harmonize identifiers, inconsistencies in protein naming conventions, accession numbers, and annotation versions across datasets may have resulted in incomplete matching. Such discrepancies could lead to an underestimation of shared differential signals between studies and potentially obscure biologically relevant relationships. Further standardization of protein identifiers and systematic mapping across database versions would likely improve the accuracy and sensitivity of cross-dataset network analyses.

Second, our analysis was restricted to publicly available labeling-based quantitative proteomic datasets. While labeling-based approaches provide relatively consistent quantification within studies, label-free quantitative data is more abundant in the repositories. The present study should therefore be regarded as an evaluation of the applicability of differential-profile integration to the labeling-based proteomic datasets analyzed here, rather than as a comprehensive assessment across all proteomic quantification strategies. Incorporating a broader range of studies could increase statistical power and enhance the discovery of novel condition-level relationships.

Future work will extend the present framework to label-free quantitative proteomic datasets. A substantial number of label-free datasets are publicly available, and adapting the directionality-based integration strategy to these data will enable evaluation of its applicability across a broader range of quantification strategies and experimental settings. Such expansion could enable larger and more diverse network constructions, potentially uncover additional biologically meaningful cross-study relationships, and help to define the conditions under which differential-profile integration is most informative.

## 5 Conclusion

In this study, we applied a differential-change–based integrative framework to proteomic data and systematically evaluated shared proteomic alteration patterns across diverse experimental conditions from quantMS and CPTAC. By emphasizing expression directionality, we demonstrated that biologically coherent relationships can be consistently extracted from large-scale datasets generated using different platforms and analytical pipelines. Analysis centered on the doxorubicin–control profile identified a distinct cluster of breast cancer samples associated with tumor stage and lipid metabolism–related pathways. These findings align with prior breast cancer studies and support the validity of the proposed approach for capturing biologically meaningful patterns in heterogeneous proteomic data. Overall, this research provides a practical, robust framework for cross-dataset proteomic data integration without relying on absolute expression levels or individual-protein statistical significance. The results provide a foundation for exploratory analyses and hypothesis generation, facilitating future validation and integrative proteomics research. We expect that the proposed strategy will encourage broader reuse of publicly available proteomic datasets and foster new insights through systematic cross-study integration.

## Supplementary Material

vbag321_Supplementary_Data

## Data Availability

The datasets analyzed in this study are publicly available. Proteomics data were obtained from the quantMS repository and the CPTAC data portal. The identifiers and accession numbers for all datasets used in this study are provided in Table S1. The code used in this research is available at https://github.com/manakanishizaki/proteome-network-integration.git. All data and code are accessible without restriction.
