# Supplementary material for: Network-based integration of cross-dataset proteomic profiles using fold-change directionality
Source: Bioinform Adv. 2026 Oct 23;6(1):vbag321. doi: 10.1093/bioadv/vbag321 (PMC13718815; doi:10.1093/bioadv/vbag321)
Supplement: Supplementary file 1 [file vbag321_supplementary_data.pdf]

Supplementary Material

Network-based integration of cross-dataset proteomic profiles using fold-change directionality

Manaka Nishizaki[1], Norie Araki[2], Shin Kawano[1,3]*

[1] Graduate School of Frontier Engineering, Kitasato University, 1-15-1 Kitazato, Minami-ku, Sagamihara-shi, Kanagawa 252-0373, Japan

[2] Graduate School of Medical Sciences, Faculty of Life Sciences, Kumamoto University, 1-1-1 Honjo, Chuo-ku, Kumamoto-shi, 860-8556, Japan

[3] Database Division for Life Science, BioData Science Initiative, National Institute of Genetics, Research Organization of Information and Systems, 178-4-4 Wakashiba, Kashiwa, Chiba 277-0871, Japan

*Corresponding author: Shin Kawano (kawano.shin@kitasato-u.ac.jp)

Supplementary Table S1. Proteomic projects included in this study.

CPTAC TMT10, CPTAC TMT11, and quantMS projects analyzed in this study are summarized. In quantMS, some datasets sharing the same ProteomeXchange accession (e.g., PXD023508) are listed separately when associated with substantially different experimental condition labels (e.g., disease, mutation carrier, and phenotype). Although derived from the same study, these datasets are treated as distinct entries.

CPTAC TMT10	CPTAC TMT11	quantMS
PDC000110	PDC000180	PDC000111
PDC000116	PDC000198	PDC000126
PDC000118	PDC000203	PXD000672
PDC000120	PDC000204	PXD002137
PDC000124	PDC000221	PXD002395
PDC000125	PDC000234	PXD003497
PDC000127	PDC000270	PXD003539
PDC000129	PDC000315	PXD004683
PDC000153	PDC000316	PXD004684
PDC000154	PDC000317	PXD004691
PDC000219	PDC000325	PXD004873
PDC000242	PDC000358	PXD010429
PDC000248	PDC000360	PXD012574
PDC000295	PDC000362	PXD014145
PDC000296	PDC000402	PXD014414
PDC000309	PDC000408	PXD014943
PDC000310	PDC000410	PXD018830
PDC000311	PDC000434	PXD020109
PDC000327	PDC000439	PXD020248
PDC000329	PDC000440	PXD021394
PDC000356	PDC000446	PXD022992
PDC000393	PDC000447	PXD023423
PDC000398	PDC000464	PXD023508-disease
PDC000400	PDC000477	PXD023508-mutation-carrier
	PDC000514	PXD023508-phenotype
		PXD025560
		PXD025864
		PXD027008
		PXD027817
		PXD028251
		PXD028618
		PXD030671
		PXD030881
		PXD032263
		PXD033169

Supplementary Figure S1

Distribution of log_2FC values by proteomic project

Project identifiers are shown above the individual box plots. Resource assignment (quantMS versus CPTAC) should be confirmed against the source plotting data before submission.

quantMS

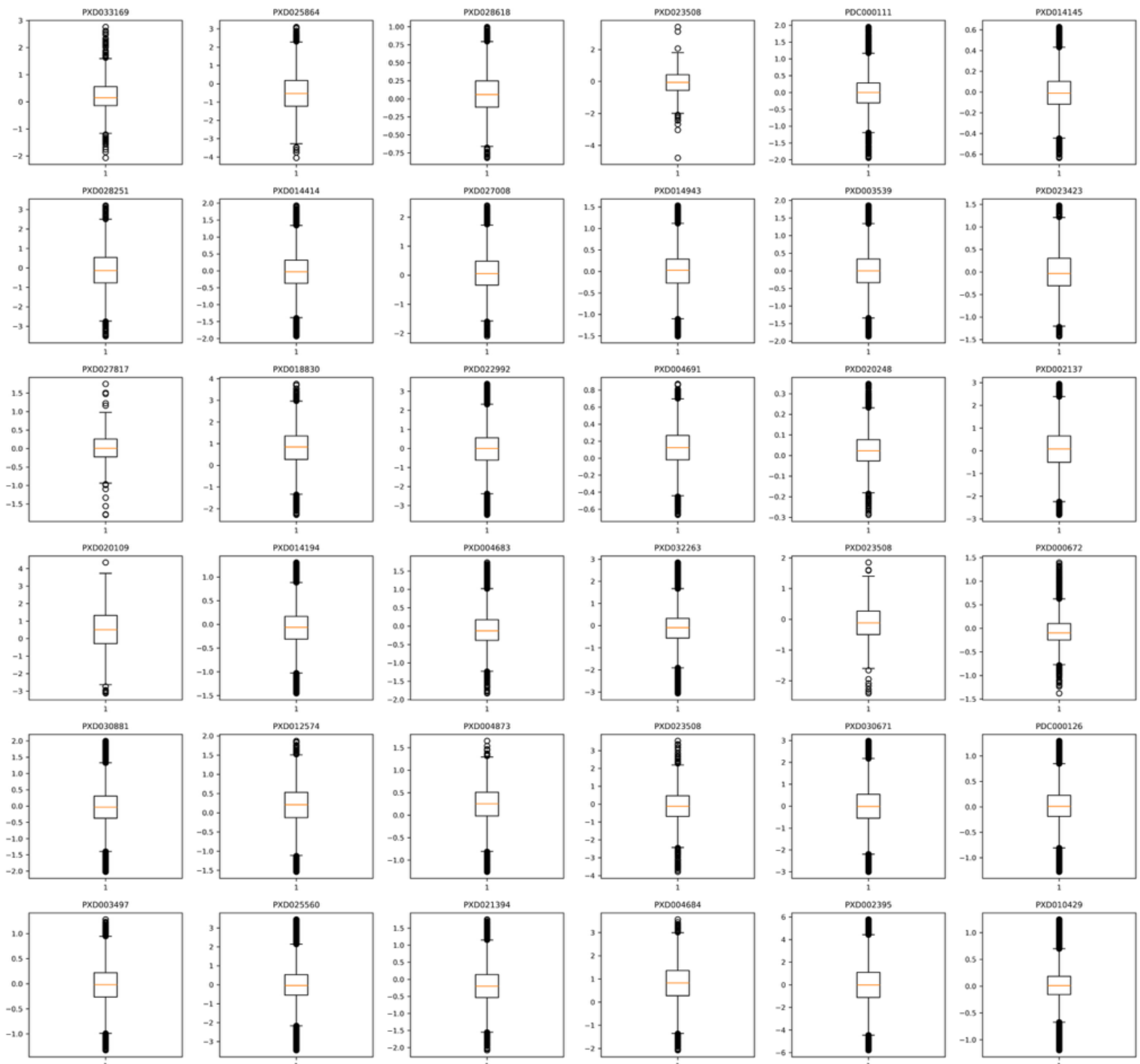

Supplementary Figure S1 (continued)

CPTAC

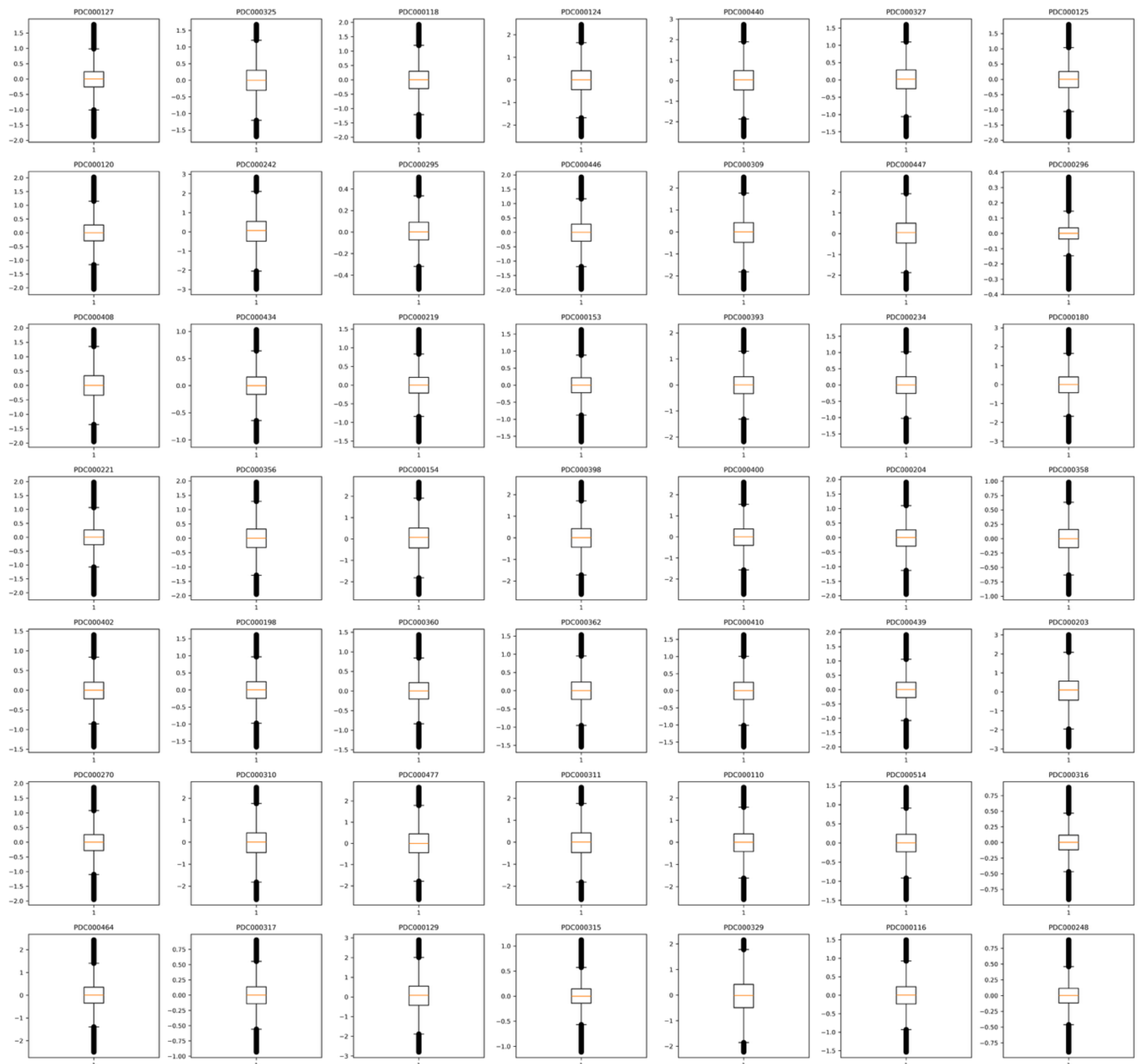

Supplementary Figure S1. Distribution of log_2FC values by proteomic project. Box plots show log2FC distributions for individual quantMS and CPTAC projects. Values exceeding ±3 standard deviations from the project-specific mean were excluded from visualization for clarity but retained in downstream analyses and quantitative datasets.

Supplementary Figure S2

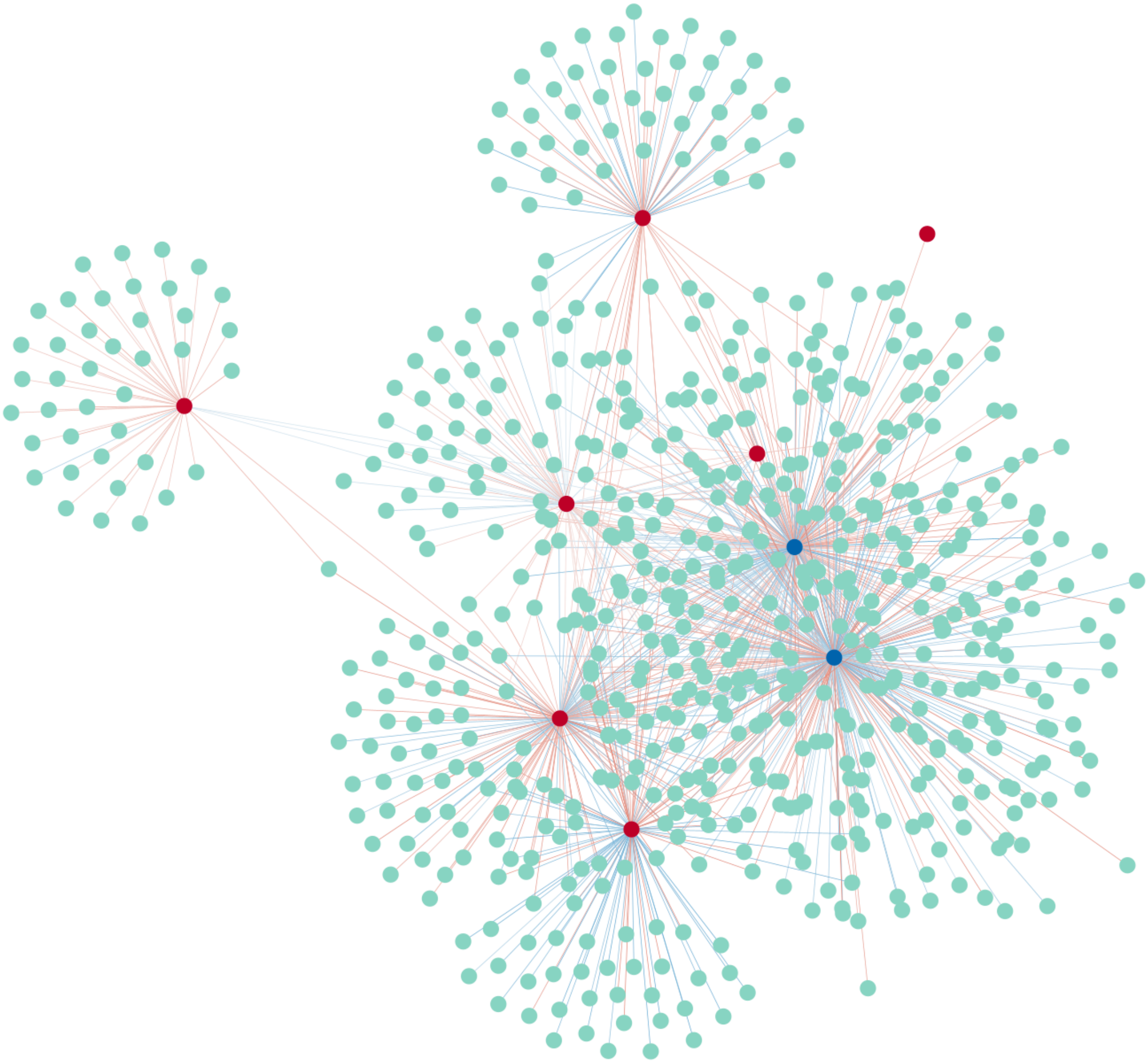

Supplementary Figure S2. Integrated network prior to excluding CPTAC datasets reanalyzed in quantMS. Network contains 639 nodes and 1,040 edges. Edge colors indicate correlation direction: red, positive; blue, negative. Network was generated before removing CPTAC-derived data included in quantMS. Hub nodes are highlighted in red, whereas nodes of interest (from PDC000126) are shown in blue.

Supplementary Figure S3

A

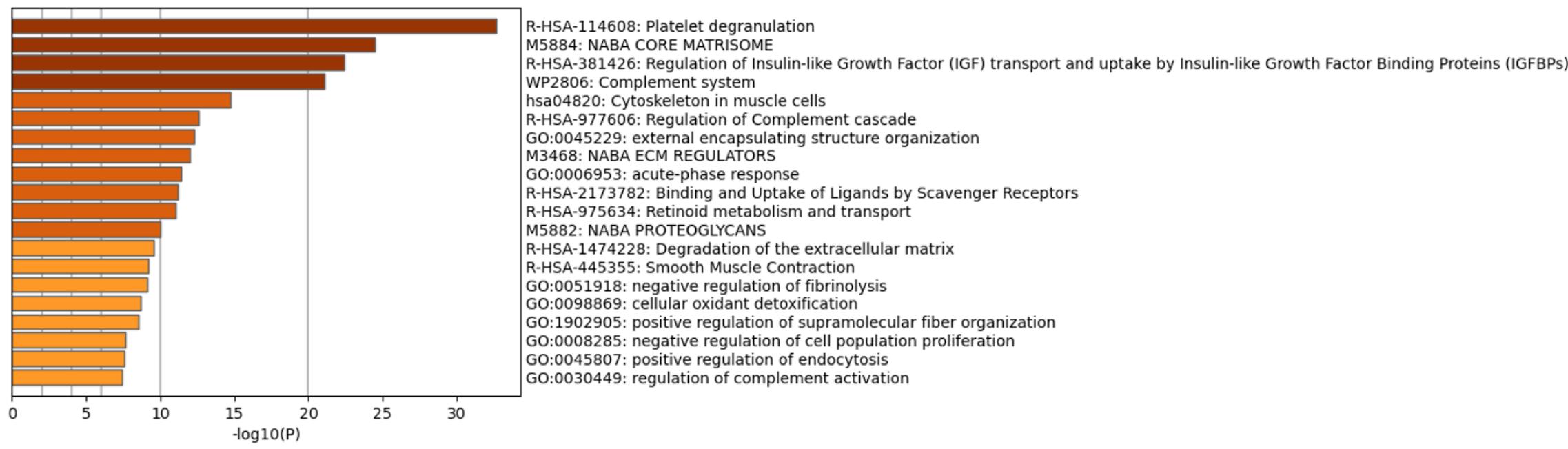

B

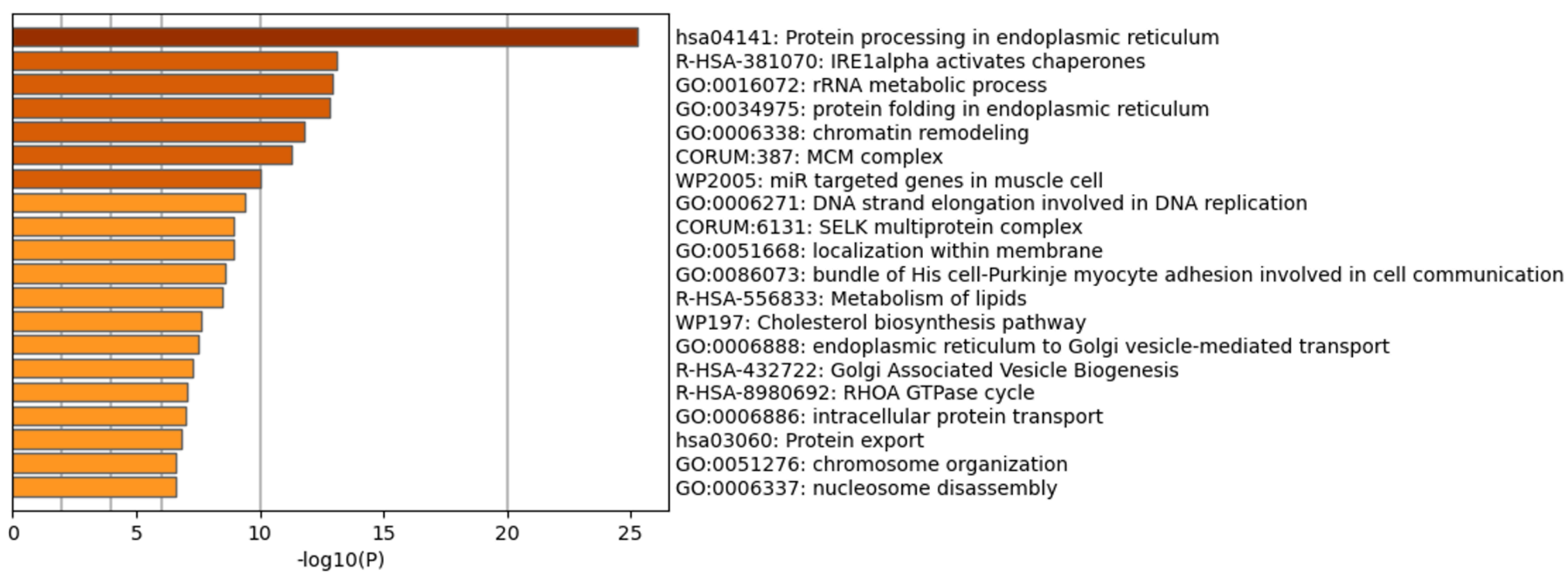

Supplementary Figure S3. Functional enrichment analysis of representative protein clusters. GO and KEGG pathway enrichment analyses were performed using Metascape for representative protein clusters showing increased (a) or decreased (b) abundance. Enriched terms are ranked by according to statistical significance (–log10 P value).
